# Immune Effects of Macrophages in Rheumatoid Arthritis: A Bibliometric Analysis From 2000 to 2021

**DOI:** 10.3389/fimmu.2022.903771

**Published:** 2022-09-12

**Authors:** YunLing Xu, Zhongmin Zhang, Jiaolong He, Zhenxing Chen

**Affiliations:** ^1^ Department of Basic Medical, Zhejiang Academy of Traditional Chinese Medicine, Hangzhou, China; ^2^ College of Pharmacy, Guangxi University of Chinese Medicine, Nanning, China; ^3^ Department of Intensive Care, First Affiliated Hospital of Jishou University, Jishou, China

**Keywords:** rheumatoid arthritis, macrophages, bibliometrics, hotspots, research status

## Abstract

Rheumatoid arthritis (RA) is a chronic autoimmune inflammatory disease characterized by macrophage activation. The current characteristics, hotspots, and research frontiers of macrophage-related RA were analyzed using bibliometric analysis. Relatedpapers published from 2000 to 2021 in the Web of Science database were retrieved. The diagrams were generated and analyzed using the bibliometric software package. VOSviewer and CiteSpace were used to evaluate and visualize the research trends and hotspots in macrophage-related RA. A total of 7253 original articles were obtained. Global research on macrophage-related RA is in an advanced stage of development, with core authors, teams and research institutions emerging. United States has published the most papers, received the most citations, and had the highest H-index over the last 22 years. The University of Amsterdam and the journal of *Arthritis and Rheumatism* are the most productive research institutions and journals. Tak PP’s (St Vincent’s Hospital) paper has the highest publication and citation scores. The keywords “bone loss” and “polarization” have the highest frequency. Additionally, the study of macrophage polarization in RA has been research focus in recent years. This study demonstrates that research on macrophages in RA will continue. China is a significant producer, whereas the United States is an influential nation in this regard. In the last decade, most studies have concentrated on fundamental research. Recent studies have shown how macrophages play a role in controlling and weakening inflammation, and drug delivery and mechanism have come to the fore.

## Introduction

Rheumatoid arthritis (RA) is an autoimmune disease associated with macrophages that manifests as a chronic inflammatory condition marked by severe joint damage and synovial joint destruction (1, 2). RA has a global prevalence of 0.3%–4.2% and is a significant global public health challenge (3, 4). Currently, the etiology of RA has not yet been fully clarified, and the disease cannot be cured entirely (5, 6). Macrophages are dynamic cells that contribute significantly to immune surveillance and respond to a variety of external stimuli (pathogenic microbes, damaged tissues, abnormal cells, etc.) (7, 8). They are abundant at the the synovium cartilage pannus junction during inflammation and are involved in the pathogenesis of RA. Macrophages are highly diverse and plastic, and they can be divided into two types: classically activated macrophage (M1) and alternatively activated macrophage (M2), each with distinct functional phenotypes (9). Thus, it is critical to quantitative analysis of the current state, its focus fields, and prospects for research on macrophage-related RA (10). Additionally, the field’s hotspots and trends are updated continuously with the introduction of new technology and clinical diagnostic standards. While several scholars have made significant contributions to this field and published numerous papers, concise summaries are lacking. As a result, a comprehensive review and summary of this field are required to benefit the research participants.

Bibliometrics is a method for assessing and monitoring the progress of specific disciplines *via* statistical analysis of published data (11). Bibliometric analysis can be used to determine the outputs and citations of countries, institutions, and authors and the keyword frequency of research hotspots and frontiers in particular fields (12). Using bibliometric analysis techniques, a 22-year (2020 – 2021) longitudinal analysis was conducted to evolution the scientific literature on macrophage-related RA. The published literature was primarily analyzed using the following criteria: publication year, country, affiliation, journal, author, keyword, citation, and H-index. Finally, the bibliometric analysis results were combined with a traditional review conducted under the guidance of bibliometrics to demonstrate the evolution of the research on macrophage-related RA. This is the first attempt to conduct an in-depth to statistical analysis of literature on macrophage-related RA. In addition, it is supposed to give reliable data that can be used to inform experimental tactics and provide useful statistics for financing decisions.

## Methods

### Data collection

The Science Citation Index (SCI) Expanded Database of the Web of Science (WoS) was used to obtain bibliographic data. To avoid bias caused by daily database updates. All documents published between 2000 and 2021 were retrieved and downloaded from the WoS Core Collection (WoSCC) database on January 19, 2022. The search strategy applied was Title = (rheumatoid arthritis OR RA) AND Title = (macrophage OR macrophages). Only English-language research papers and review articles were retrieved. Two investigators (YLX and JLH) independently conducted the primary data search and discussed any discrepancies. The final agreement reached a value of 0.90, indicating a substantial agreement (13).

**Figure 1:**
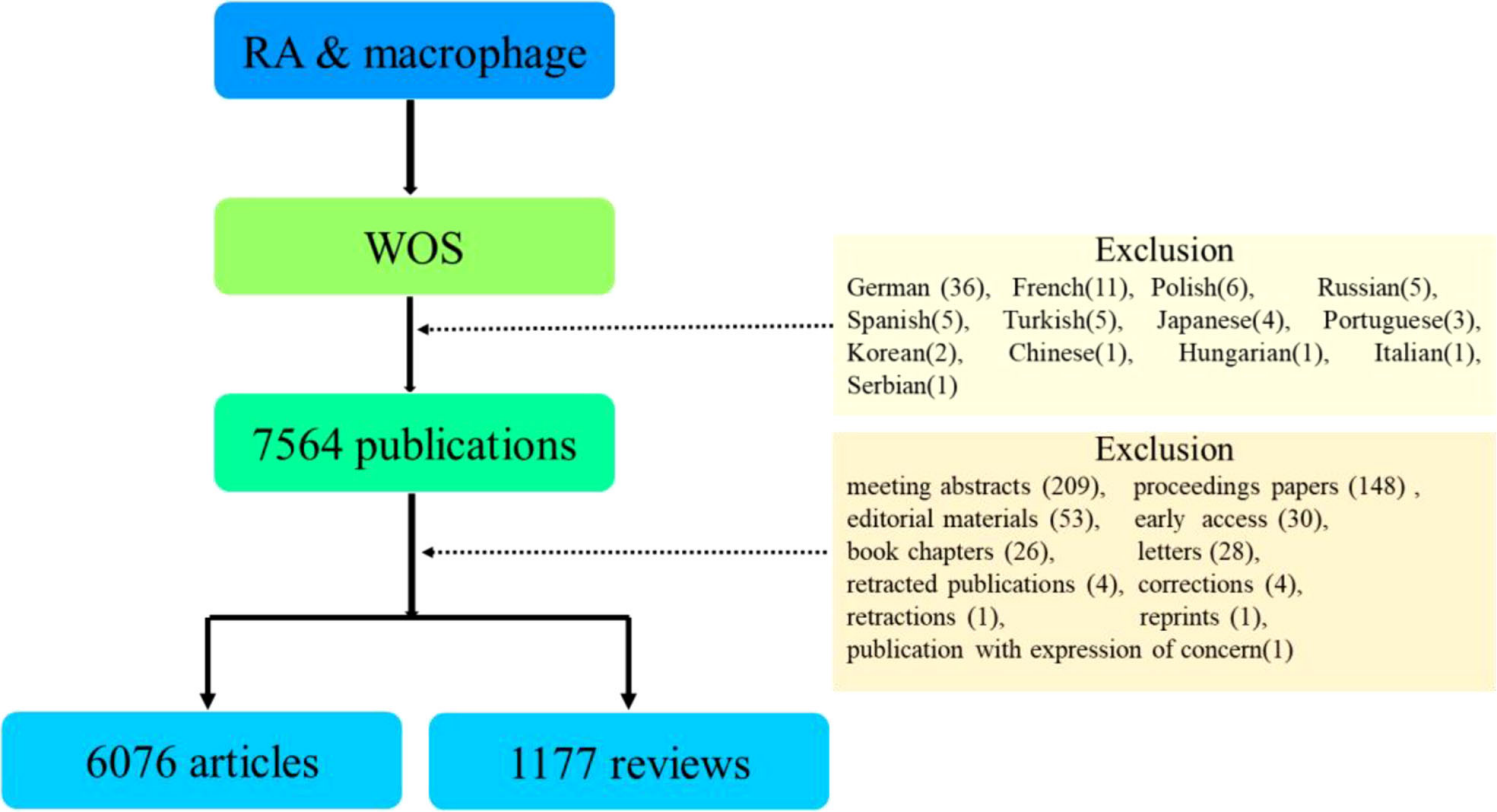
Flowchart of literature selection.

The data was saved and stored in download_txt format. For further analysis, only research articles and review articles chosen, as data acquisition flowchart presented in **Figure 1**. This study excluded abstracts from meeting, proceeding papers, editorial materials, early access, letters, book chapters, corrections, retracted publications, publication with expression of concern, reprints, and retractions.

### Bibliometric analysis

For visual analysis, all valid data collected from the WoSCC database was imported into CiteSpace (version 5.7) and OVSviewer (version 1.6.16) (14). CiteSpace was used to analyze the strongest citation bursts of references and keywords, investigate the research status, hotspots, and trend distribution maps over time, and determine the field’s development trend (15). The collaborative networks between countries, institutions, journals and authors and co-citation of keyword clusters were visually analyzed using VOSviewer. The research status, hot spot and development trend in this field can fully understand by examining keyword frequency, centrality intensity, and prominence. A co-word network was constructed based on the co-occurrence of keyword co-occurrence, with each node representing a keyword. When two keywords appear in the same article, they form a co-occurrence relationship and are represented in the network as a single edge. A large mean value indicates that the node has significant representation in a particular subject field at a specific time. The degree of emergence suggests how much a node’s collinear frequency and the number of co-citations increase over time. The greater the degree of emergence, the more evidence that the node was a research hotspot during a given period.

## Results

### Annual publication outputs

**Figure 2:**
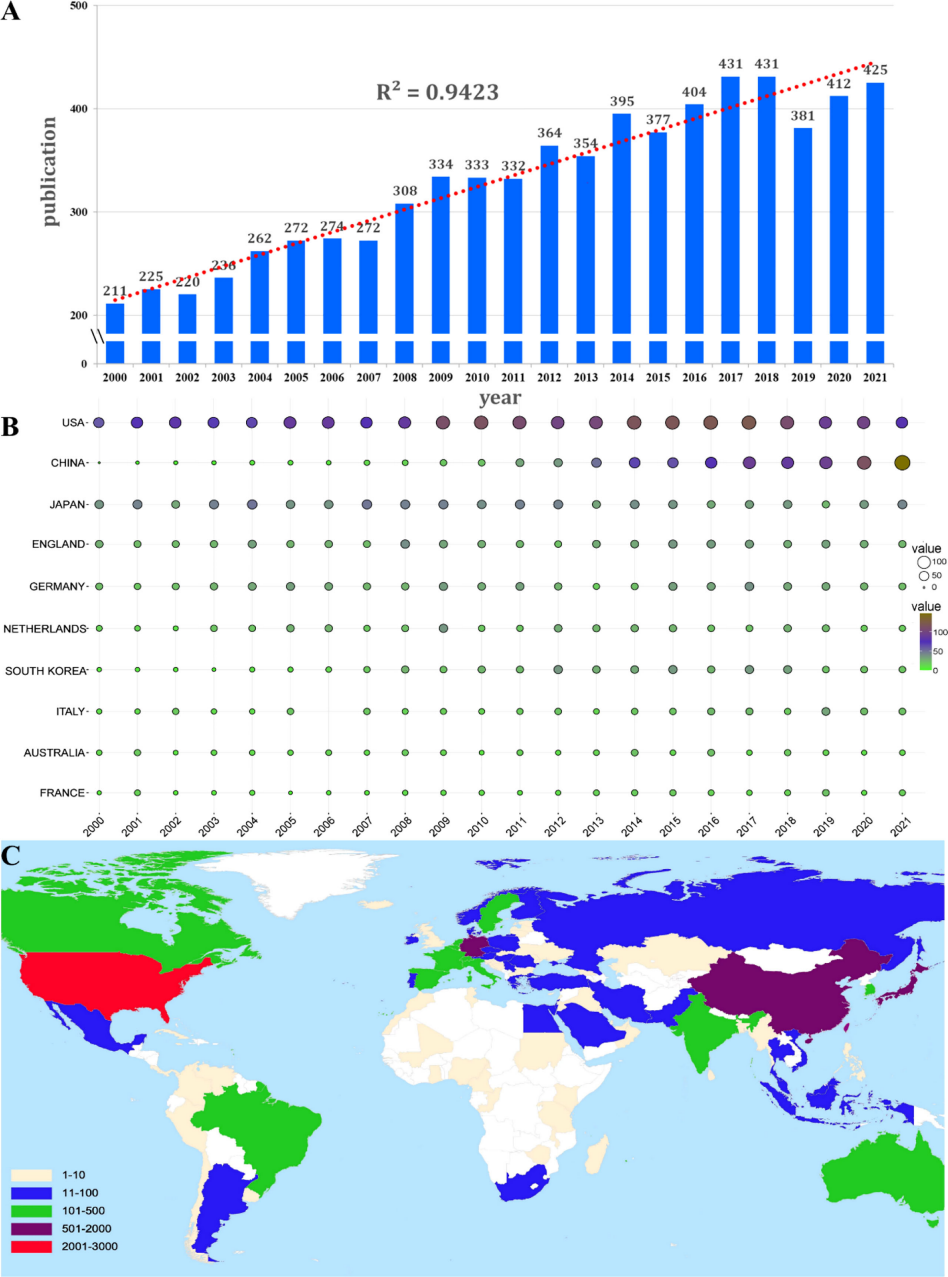
**(A)** The number of publications by year and curve fitting of the annual growth trend of publications (R^2^ = 0.9423). **(B)** The annual number of publications in the top 10 most productive countries from 2000 to 2021. **(C)** Global distribution according to the country publications.

The total number of publications (NP) over a given period can objectively and quantitatively reflect a field’s overall development trend. A total of 7253 publications were chosen based on the defined search terms. Among these publications, 6076 (83.77%) were original articles, and 6076 (16.23%) were reviews. The annual NP is depicted in **Figure 2A**. Despite some fluctuations, the NP increased from 211 in 2000 to 425 in 2021. Over the last 22 years, the growth rate has been relatively stable. **Figure 2A** also depicts a polynomial fitting curve for the publication’s total annual growth trend. The annual NP trended upward and was highly correlated with the year of publication (R^2^ = 0.9423). Overall, these findings indicate that the research on macrophage-related RA has gradually stabilized.

### Distribution of countries/regions and institutions

The publications originated from 103 countries/regions, and 64 countries/regions had more than four publications. The top 10 most influential countries/regions are listed in **Table 1**, along with their NP, the total number of citations (NC), H-index, and average citation per item (AC). The top 10 countries/regions published 96% (7018/7253) of the publications. The United States was the leading country in terms of NP (28.7%,2079/7253), followed by China (13.2%,958/7253) and Japan (11.9%,866/7253). The top 3 countries with the highest NC were the United States (129061), England (38338), and Japan (36890). The top 3 countries with the highest AC were the United States (63.90), France (63.63), and the Netherlands (61.57). **Figure 2B** shows the top 10 most productive countries’ annual NP from 2000 to 2021. The circle’s size and colors correspond to the NP and citation values of the papers, respectively. Research on macrophage-related RA in the United States is at the core of global studies, and China’s research has been more active in recent decades. **Figure 2C** depicts the global distribution of publications by country, and **Supplementary Figure 1** contains the countries VOSviewer visualization map.

**Table 1:** Top 10 most productive country in macrophages-related RA from 2000–2021.

Rank	Country	NP	NC	H-index	AC
1	USA	2079	129061	160	63.90
2	China	958	18191	62	19.76
3	Japan	866	36890	93	43.36
4	England	636	38338	99	61.47
5	Germany	613	32143	90	53.31
6	Netherlands	478	28419	93	61.57
7	South Korea	455	14087	60	31.50
8	Italy	384	21187	74	56.04
9	Australia	300	15034	71	52.16
10	France	249	15714	64	63.63

NP, total number of publications; NC, total number of citations; AC, Average Citations per Item.

**Figure 3:**
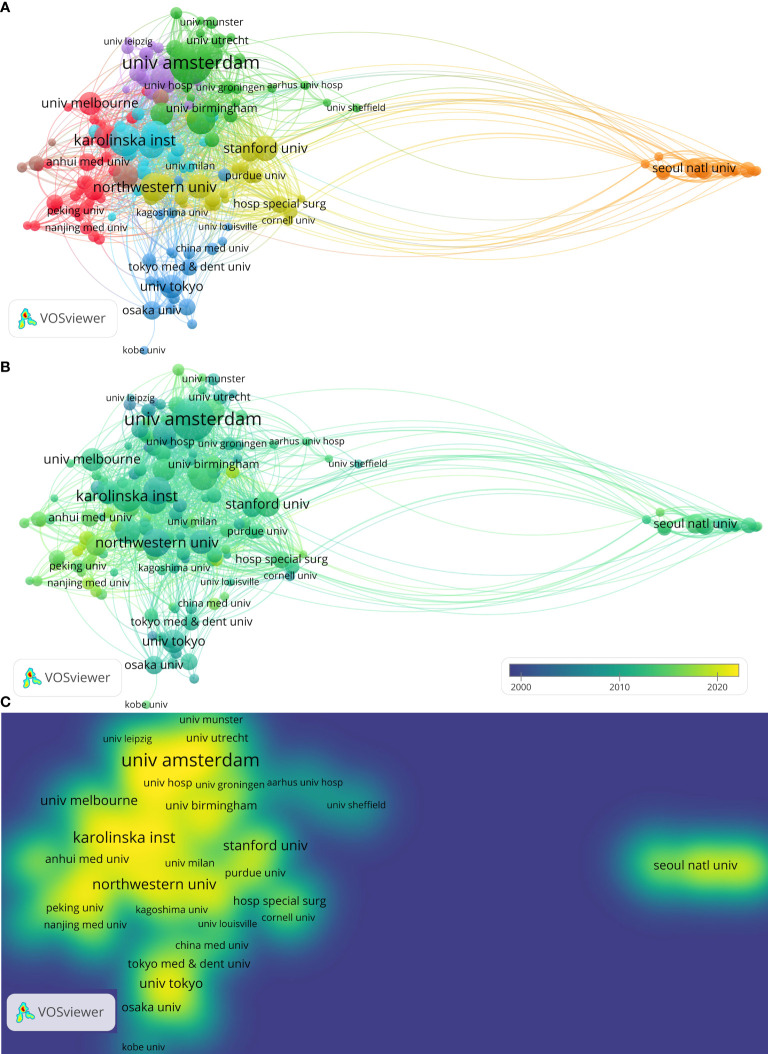
Cluster analysis of publications from different institutions from 2000-2021. **(A)** Cluster analysis of publications from different institutions. **(B)** Evolution of publications from different institutions. **(C)** Evolution of publications from different institutions frequency.

More than 5409 institutions have made contributions to this field, with 161 producing more than 18 papers. The visual cluster analysis (threshold > 18 papers) result is depicted in **Figure 3**. The 161 institutions that appeared more than 18 times were color-coded into eight clusters. A larger node indicated that more documents had been transmitted, and the largest node was highlighted in a green. The line illustrates the connection between the institutions. By and large, the institutions were concentrated around universities, and only a few were hospitals. According to the clustering results, University of Amsterdam, Karolinska Institutet, Leiden University, Imperial College of Science, University of Oxford, University of Glasgow, Northwestern University, Harvard University, University of Genoa, and the University of Tokyo collaborated and exchanged research on macrophages-in RA. **Table 2** summarizes the top 10 most influential institutions. The institutions with the highest NP values were the University of Amsterdam (165), followed by the University of London (157), and the University of Oxford (154). The most significant AC scores were from Karolinska Institute (AC = 73.08), Harvard University (AC = 71.56), and Institut National de la Sante et de la Recherche Medicale (AC = 68.12).

**Table 2:** Top 10 productive affiliations in macrophages-related RA from 2000–2021.

Rank	Affiliations	NP	Country	NC	H-index	AC
1	University of Amsterdam	165	Netherlands	9511	61	60.24
2	University of London	157	England	9510	55	61.04
3	University of Oxford	154	England	9511	57	62.76
4	University of California System	145	USA	9342	55	64.65
5	Harvard University	142	USA	10118	55	71.65
6	Institut National De La Sante Et De La Recherche Medicale	138	France	9338	50	68.12
7	Academic Medical Center Amsterdam	137	Netherlands	8907	60	67.77
8	Karolinska Institutet	128	Sweden	9258	46	73.08
9	Us Department of Veterans Affairs	125	USA	5376	45	44.20
10	Veterans Health Administration	122	USA	5259	44	44.24

NP, total number of publications; NC, total number of citations; AC, Average Citations per Item.

### Funding source

Funding support plays a vital role in scientific advancement. **Table 3** summarizes the top 10 funding agencies and sponsors in this field. In the terms of funding, the United States’ research funding agencies, including the Department of Health Human Services, the National Institutes of Health, the National Institute of Arthritis Musculoskeletal Skin Diseases, the National Institute of Allergy and Infectious Diseases, the National Heart Lung Blood Institute, and the NIH National Cancer Institute, occupied the primary positions that promoting to macrophage-related RA research. The remaining funds came from the China’s National Natural Science Foundation, the European Commission, Japan’s Ministry of Education Culture Sports Science and Technology and the United Kingdom’s Research Innovation. Along with established institution the United States has maintained at the forefront of research into macrophage-related RA research due to having sufficient funding.

**Table 3:** The top 10 funding source with most publications.

Rank	Funding source	NP	NC	H-index	AC
1	United States Department of Health Human Services	1,179	81689	134	70.69
2	National Institutes of Health (NIH)	1,176	81310	133	70.52
3	National Natural Science Foundation of China (NSFC)	510	8787	46	17.86
4	European Commission	497	27207	85	55.32
5	NIH National Institute of Arthritis Musculoskeletal Skin Diseases (NIAMSD)	463	32255	96	71.43
6	NIH National Institute of Allergy and Infectious Diseases (NIAID)	337	24590	86	73.85
7	NIH National Heart Lung Blood Institute (NHLBI)	260	21377	75	82.63
8	Ministry of Education Culture Sports Science and Technology Japan Mext	235	6416	42	27.55
9	UK Research Innovation (UKRI)	173	10955	57	63.96
10	NIH National Cancer Institute (NCI)	168	12786	58	76.38

NP, total number of publications; NC, total number of citations; AC, Average Citations per Item.

### Authors and co-citation authors

Over 36894 authors have authored in this field. Among them, 207 authors contributed at least eight papers to this collection. **Table 4** summarizes the top 10 most productive authors. Tak PP of St. Vincent’s Hospital was the most prolific author (NP = 110, NC = 7382) with the highest H-index (55), and McInnes IB from the University of Glasgow had the most increased AC (94.47). A relationship of co-citation has been established between two documents that appear in the references of a third document (16). Co-cited authors are two or more authors who are simultaneously cited in one or more papers. These authors are related *via* co-citations. Among the 120146 cited authors, 140 (classified into seven clusters) had at least 106 citations. Based on the cluster summary, the authors devoted their minds to summarizing the evolving concepts, pathogenesis, related outcomes, and interventions of RA. Firestein GS ranked the highest in NC (798), followed by McInnes IB (770), Feldmann M (758), Tak PP (576), and Smolen JS (559). **Supplementary Figures 2**, **3** contain the co-authorship authors’ and co-citation authors’ VOSviewer visualization maps.

**Table 4:** The top 10 active authors in macrophages-related RA from 2000–2021.

**Rank**	**Author**	**Affiliations**	**Country**	**NP**	**NC**	**H-index**	**AC**
1	Tak PP	St Vincent’s University Hospital	Ireland	110	7382	55	70.45
2	Van Den Berg WB	Radboud University Nijmegen Medical Centre	Netherlands	59	3766	32	65.46
3	Bucala R	Yale University	USA	55	3374	35	64.85
4	Pope RM	Northwestern University	USA	48	2794	30	61.65
5	Li J	University of Alabama at Birmingham	USA	47	1619	23	34.64
6	Wang Y	Anhui Medical University	China	47	1211	21	25.98
7	McInnes IB	University of Glasgow	Scotland	45	4209	29	94.47
8	Cutolo M	University of Genoa	Italy	44	2235	29	52.64
9	Zhang Y	Mudanjiang Medical University	China	41	1141	18	27.98
10	Feldmann M	Imperial College of Science, Technology and Medicine	England	40	2715	26	68.8

NP, total number of publications; NC, total number of citations; AC, Average Citations per Item.

### Source journals and co-citation journals

A total of 1382 journals published specific articles on macrophage-related RA. Among them, 160 journals contributed at least eight papers. **Table 5** lists the top 10 most productive journals, their publishers, NP, impact factor (IF), NC, and H-index. Most of the journals specialize in arthritis and immunity. Four publishers are based in the the United States, while three are in England. *Arthritis and Rheumatism* was the most prolific journal (NP=347) with the highest NC (27563), and AC (80.76). *Annals of the Rheumatic Diseases* had the highest IF (27.973), followed by *Arthritis and Rheumatology* (15.483), respectively.

**Table 5:** The top 10 most productive journals.

Rank	Journal	Country	ISSN	NP	IF (2021)	NC	H-index	AC
1	Arthritis & Rheumatology	United States	2326-5191	347	15.483	27563	95	80.76
2	Arthritis Research & Therapy	England	1478-6354	287	5.606	13351	62	47.13
3	Journal of Immunology	United States	0022-1767	257	5.426	19813	84	77.40
4	Annals of the Rheumatic Diseases	England	0003-4967	199	27.973	12891	70	65.48
5	Frontiers in Immunology	Switzerland	1664-3224	161	8.786	4490	35	28.17
6	PLoS One	United States	1932-6203	138	3.752	3352	33	24.36
7	Journal of Rheumatology	Canada	1499-2752	126	5.346	4796	42	38.32
8	Rheumatology	England	1462-0324	121	7.046	6840	49	56.90
9	International Immunopharmacology	Netherlands	1567-5769	82	5.714	1854	25	22.80
10	Scientific Reports	England	2045-2322	81	4.996	1776	25	20.38

NP, total number of publications; IF, Impact factor; NC, total number of citations; AC, Average Citations per Item.

Co-cited journals are those in which two or more journals are cited concurrently by researchers. The threshold was met by 141 of met the 12023 cited references (minimum citations > 400). The *Journal of Immunology, Arthritis and Rheumatism*, the *Journal of Biological Chemistry*, *Journal of Experimental Medicine*, and *Annals of the Rheumatic Diseases* were the five most frequently and centrally cited journals. **Supplementary Figure 4** illustrates the VOSviewer visualization map of the co-cited journals.

### Citations documents and journals

**Figure 4:**
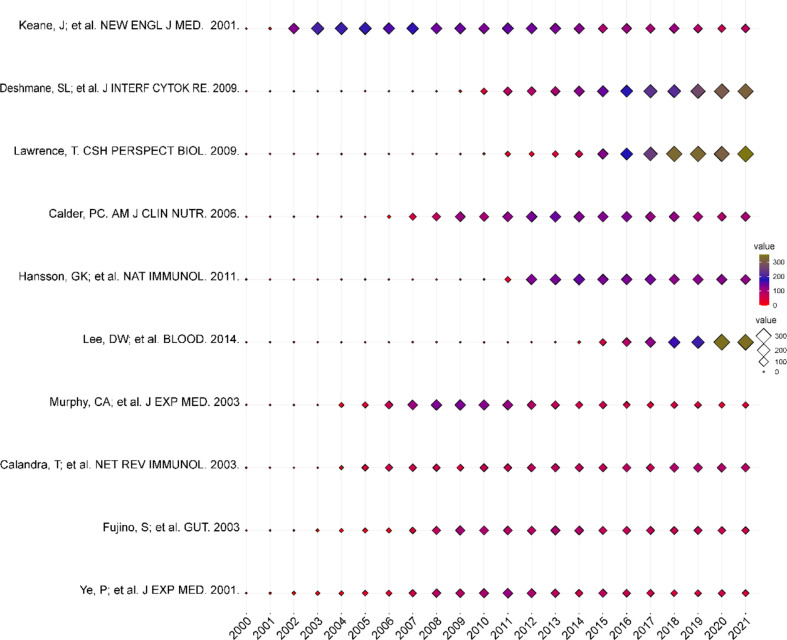
The yearly number of citations of papers with high citations.

Of the 7253 documents, 384 met the threshold of minimum citations >145. The top 10 articles with the highest NC are presented in **Figure 4**. The highest NC of the paper written by Joseph Keane in 2001 was 2681, ranking it first followed by Satish L. Deshmane’s (NC =2097) and Toby Lawrence’s (NC = 2010). In Joseph Keane’s paper, the authors summarized the reports of Crohn’s disease and RA after tumor necrosis factor α (TNF-α) neutralizing agent infliximab treatment(17). Furthermore, Satish L. Deshmane’s work identified of monocyte chemoattractant protein-1 (MCP-1/CCL2) as one of the key chemokines that regulate monocytes/macrophages migration and infiltration. It summarized their biological processes and the structure and function of CCL2 (18). Toby Lawrence described how chronic inflammatory diseases like rheumatoid arthritis are linked to nuclear factor-kappa B (NF-κB) activation (19). He reveals complex roles for the NF-κB in inflammation that suggest both pro- and anti-inflammatory roles for this pathway. These documents made a big difference in studying macrophages in RA and could be called “seminal”, which led to more research in this field.

Of the 1382 documents sources, 103 met the criterion (minimum number of documents of a source > 12). *Arthritis and Rheumatism* was ranked first in the terms of publications and citations (NP= 347, NC= 26634), follow by *Arthritis Research and Therapy* (NP=287, NC= 12435) and *Annals of the Rheumatic Diseases* (NP= 199, NC = 12633). **Supplementary Figures 5**, **6** show the VOSviewer visualization map of citations to document and journals in this field.

### References citation bursts

**Figure 5:**
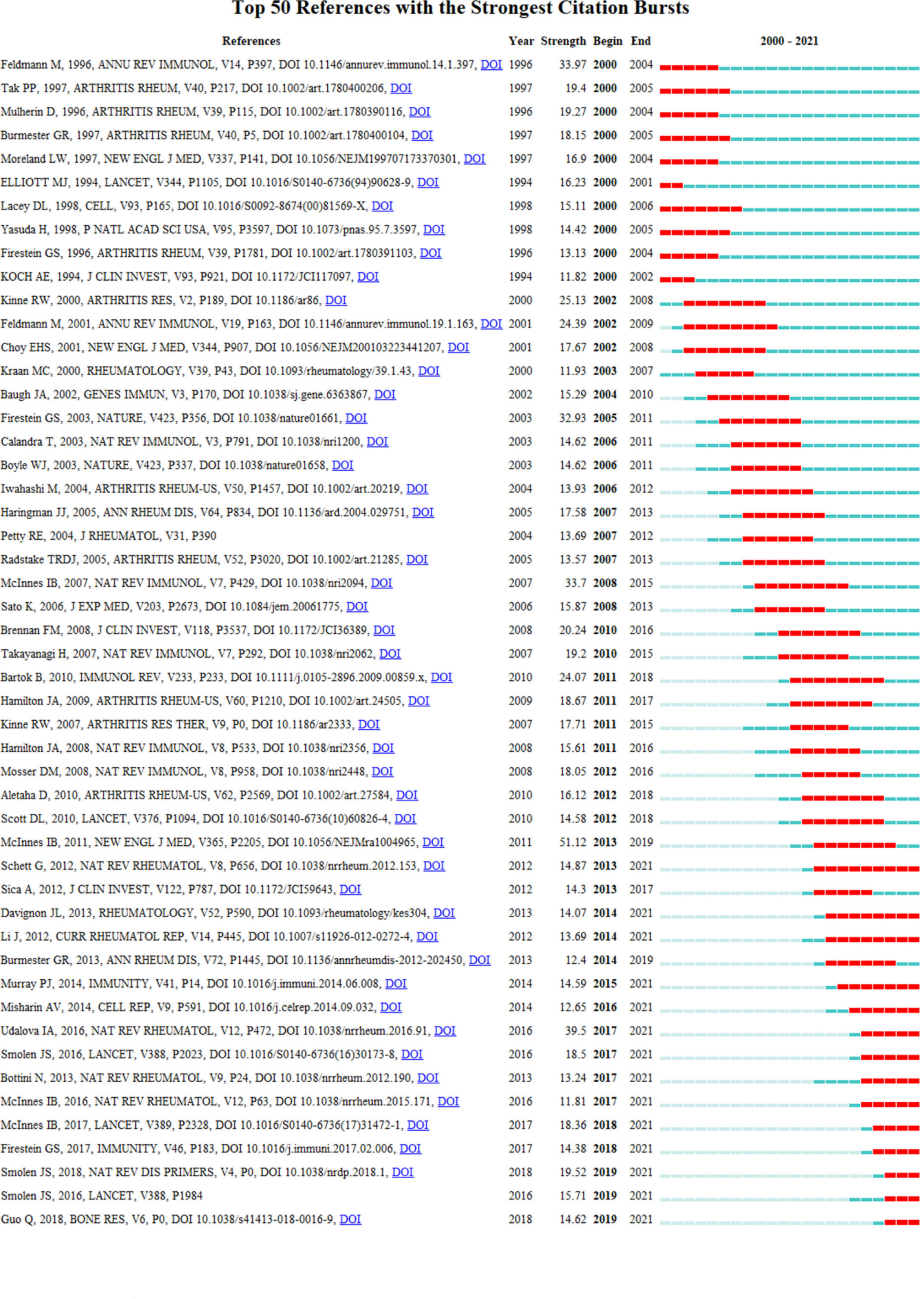
Visualization map of top 50 references with the strongest citation bursts involved in macrophage-related RA.

Burst detection, an algorithm developed by Kleinberg (20), is an effective analytic tool for capturing rapid increases in the popularity of references or keywords over a specified time period. This function can quickly identify concepts or topic that are actively discussed during a specified period. The present study applied the burst detection algorithm to extract key references and keywords for macrophage-related RA research. **Figure 5** illustrates the top 50 references with the strongest citation bursts. The blue lines represent the time period in this graph, and the red lines represent the period when the reference burst occurred. Among these references, four reference with the strong burst value was written by McInnes IB et al. In his articles summarized the crucial effector functions of immunological processes and key effector functions of cytokines in the pathogenesis of RA (21). Then give a conspectus of that macrophage-derived cytokines (TNF-α, IL-1β, IL-6, IL-12 and IL-18) activate multiple pro-inflammatory pathways in synovial tissue and responsible for osteoclasts maturation and activation (22, 23). Furthermore, in RA patients’ cytokines promote joint inflammation and destroy immunity and articular cartilage. NF-κB ligand receptor activator (RANKL), TNF, IL-17 and IL-1 play a graded role in this process.

### Keyword co-occurrence and burst

**Figure 6:**
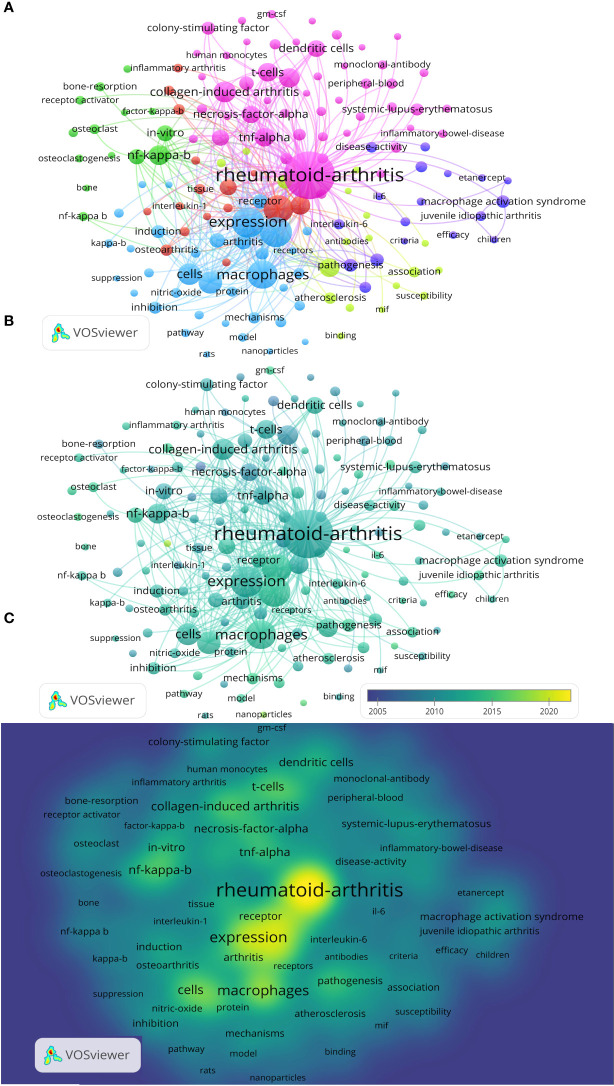
Cluster analysis of keywords from 2000-2021. **(A)** Cluster analysis of keywords. **(B)** Evolution of keywords. **(C)** Evolution of keywords frequency.

Keyword analysis can also determine the time when keyword with a frequency change rate first appeared in a node, thereby defining the the research field’s boundaries. Between 2000 and 2021,19174 keywords were used, with 140 of them appearing more than 69 times. These studies look at RA’s clinicopathological diagnosis, RA related-inflammatory markers, the pathogenic mechanisms of RA, the therapeutic interventions, and how drugs work to treat RA. As illustrated in **Figure 6**, the top 10 keywords were “rheumatoid-arthritis”, “expression”, “inflammation”, “macrophages”, “activation”, “collagen-induced arthritis”, “NF-κB”, “cytokines”, “TNF-α”and “T cell”. Among these keywords, the most concerned macrophage-derived cytokines are is TNF, other such as IFN-γ, colony-stimulating factor (CSF), IL-6, nitric oxide (NO), chemokines, growth-factor-beta (GF-β), migration inhibitory factor (MIF), lipopolysaccharide (LPS), endothelial growth-factor, granulocyte/macrophage colony stimulating factor (GM-CSF), monocyte chemoattractant protein-1(MCP-1), interleukin-1 (IL-1), proinflammatory cytokines, cyclooxygenase-2 (COX-2) have also attracted the attention of researchers (occurrences > 60, **Table 6**). Moreover, the high frequency keywords of RA-related macrophage function were: cytokines, activation, differentiation, apoptosis, chemokines, cytokine production, innate immunity, oxidative stress, immune-response, polarization, migration inhibitory factor, proinflammatory cytokines, metabolism, signal-transduction, migration (occurrences > 60, **Table 6**).

**Table 6:** Top keywords of macrophage-derived cytokines and RA-related macrophage functions (occurrences> 60).

Rank	Macrophage-derived cytokines		RA-related macrophage functions	
	Keyword	Occurrences	Keyword	Occurrences
1	tumor-necrosis-factor (TNF-*α*)	1786	cytokines	825
2	interferon-gamma (IFN-*γ*)	288	activation	819
3	colony-stimulating factor (CSF)	259	differentiation	344
4	interleukin-6 (IL-6)	244	apoptosis	229
5	nitric oxide (NO)	231	chemokines	199
6	chemokines	199	cytokine production	128
7	growth-factor-beta (GF-*β*)	170	innate immunity	114
8	migration inhibitory factor (MIF)	165	oxidative stress	114
9	lipopolysaccharide (LPS)	123	immune-response	75
10	endothelial growth-factor	107	polarization	72
11	GM-CSF	107	migration inhibitory factor	70
12	monocyte chemoattractant protein-1(MCP-1)	100	proinflammatory cytokines	68
13	interleukin-1 (IL-1)	94	metabolism	65
14	proinflammatory cytokines	68	signal-transduction	64
15	cyclooxygenase-2 (COX-2)	63	migration	63

**Figure 7:**
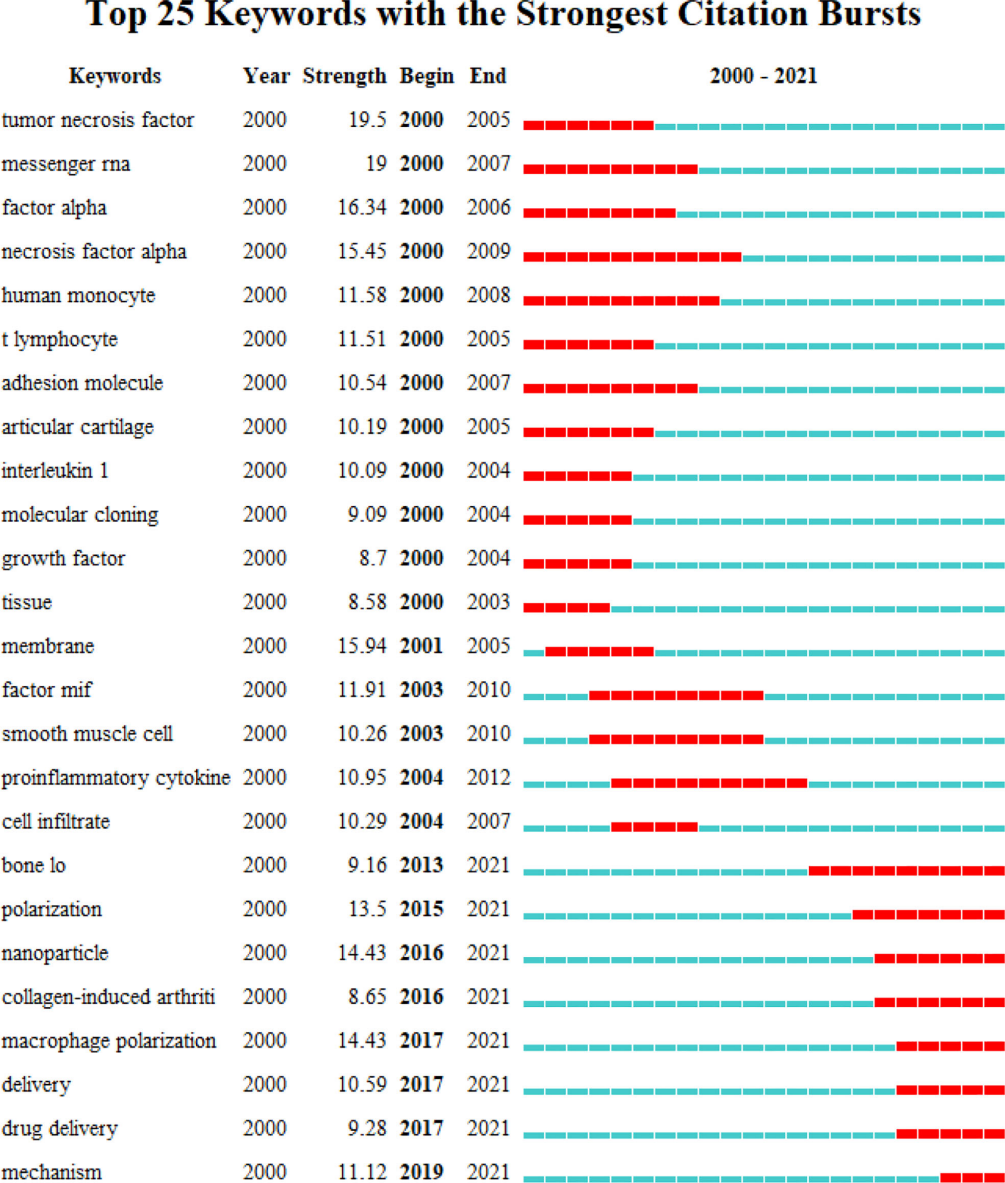
CiteSpace visualization map of top 25 keywords with the strongest citation bursts involved in macrophage-related RA.

“Burst words” are words that are frequently cited in connection with a particular subject at a specific time. They enable the observation of emerging theories and the prediction of the research frontier base on the distribution of keywords with the highest citation burst. **Figure 7** lists the keywords with the strongest citation bursts. As shown, the keywords with strong bursts before 2013 were “TNF”, “mRNA”, “factor alpha”, “necrosis factor-alpha”, “human monocyte”, “T lymphocyte”, “adhesion molecule”, “articular cartilage”, “IL”, “molecular cloning”, “growth factor”, “tissue”, “smooth muscle cell”, “proinflammatory cytokine”, “cell infiltrate”, “membrane” and “factor MIF”. While the burst keywords after 2013 included “bone loss”, “polarization,” “nanoparticle”, “collagen-induced arthritis”, “macrophage polarization”, “delivery,” “drug delivery” and “mechanism”, which represent the emerging trends.

## Discussions

### General information of macrophage-related RA research

A bibliometric analysis was performed to investigate the developmental trends and hotspots of research on macrophage-related RA from the SCI-Expanded database using VOSviewer and CiteSpace. A total of 7253 original articles and reviews published from 2000 to 2021 were obtained. According to the polynomial fitting curve, the annual NP was generally upward, but the growth stalled in the second half of the curve, particularly after 2015. The lack of high-impact, ground breaking publications was the main reason why the annual NP didn’t move forward for recent three years (**Figure 2A**).

Among the top countries, the United States ranked first in terms of publication quality, indicating that the United States is a highly productive country in terms of macrophage-related RA (**Figures 2B**, **2C**; **Table 1**). Four United States affiliations, six grants and three United States authors ranked among the top 10 affiliations, funding source and authors in the research of macrophage-related RA (**Tables 2** **–** **4**). This result indicates that the United States possess the world’s most elite institutions, abundant funding and professional researchers, which partially explaining why the United States has developed rapidly in this field over the past 22 years. Similarly, Japan and high-income countries in Europe, such as England, Germany, the Netherlands, Italy, and France, consistently had high academic productivity and an H-index of>90 each year, indicating the countries’ continued commitment to and investment in the field. However, China’s total number of articles published has increased dramatically over the last 22 years compared to the aforementioned countries. The country is now ranked second overall, though its H-index remains low. In this case, Chinese institutions and scholars need to do more to improve the research quality and make their research better. Parallel to China’s economy development, financial support for medical research has continued to increase, which may be the one of the reasons for China’s NP growth over the last 22 years. According to these data, it is foreseeable that China’s academic productivity will get better and better, and its impact on global academic productivity will become greater in the near future.

Notably, of the top 10 most productive journals, 8 had high IF values (>5.0). This finding indicates that publishing research on macrophage-related RA in high-quality journals is not a challenging. The 50 articles with high NC were published in journals with a high-IF, indicating that these journals have published a greater number of potential breakthroughs in this field. As a result, researchers interested in this field should pay more attention to the news in these journals.

### Research foci

There are many different immune cells affect in RA development. In addition to macrophages, immune cells such as T cell, B cell, dendritic cell, mast cell, natural killer cell are also involved in immune responses (24, 25). Of course, many articles have studied the relationship between these cells (**Supplementary Table 1**). In these publications, the highest frequency of other immune cells was described in the same paper with macrophage-related RA is T cell, accounting for 44.78% (3248/7253). Compared to other chronic diseases, RA shares numerous inflammatory pathological and immunological similarities. The expression of inflammation factors by excessive macrophage activation has long been a research hotspot in RA (**Figure 6**). In RA, hyperactivated macrophages increased the expression of toll-like receptors (TLRs), such as TLR2, TLR3, TLR4, and TLR7, which induce synovial inflammation and cartilage destruction by releasing chemokines, pro-inflammatory cytokines, and degradative enzymes have been recognized (26, 27). Activation of M1 macrophages, secrete a variety of pro-inflammatory cytokines (TNF-α, IL-1β, IL-6, chemokines) and activate inducible nitric oxide synthase to produce NO. Their key mechanisms of action in promoting inflammation and bone destruction have received the most extensively research in the context of RA-related macrophage functions (**Table 6**). M2 macrophages can secrete various anti-inflammatory factors, including transforming growth factor-β (TGF-β) and IL-10, and are critical players in the regression and repair of RA inflammation (28, 29). In patients with RA, the abnormal immune microenvironment promotes metabolic reprogramming, alters macrophage polarization states, disrupts the dynamic balance of M1 and M2 macrophages, and delays tissue inflammation. Aa a result, inhibiting M1 macrophage polarization and inducing M2 macrophage polarization are ideal drug research and development strategies in the treatment of RA (30, 31).

Synovial macrophage contributes to the synovial inflammatory response *via* TNF, IL-1 and other pro-inflammatory cytokines, as well as cell-to-cell contact, aggravating RA’s disease (32). TNF is a well-established RA driver that regulates inflammation, autoimmunity, and joint destruction in RA patients’ joints (33). As the statistics show, TNF is the most watched macrophage-derived cytokines, as 1275 of these publications were related to RA-treatment targeting to TNF (**Supplementary Table 2**). TNF inhibitor biologics have emerged as beneficial treatment options for amelioration of RA, and clinical remission has become a viable therapeutic target (34). Early intervention, such as TNF blockers, can increase the rate of clinical response and the likehood of clinical symptom improvement (35, 36). Additionally, biologics targeting on IL-1, IL-6, IL-10, IL-17 and GM-CSF are also favored by researchers, and relevant clinical trials are underway (37, 38).

Currently, there is no complete cure for RA, existing treatments are limited by frequent drug administration, poor penetration across biological barriers, temporary remission, and severe long‐term adverse effects (39, 40). In recent years, researchers have also increasingly invested in the field of drug deliver, such as bio-active nanoparticles and macrophage-derived macrovesicle-coated nanoparticle to overcome these obstacles (**Figure 7**), which can accumulate in chronic inflamed tissue by increasing drug permeation, increase drug concentration in inflamed joints, and actively binding to receptors overexpressed by cells in inflamed tissues, thereby maximizing efficacy and minimizing systemic adverse effects (41, 42). They are capable of effectively targeting drugs in inflamed joints to treat RA (43, 44).

### Limitations

This study is based on bibliometric and visualization analyses, which may provide important evidence to the current research state and overall trend on the field’s research academic frontiers. Additionally, this study employs NC as an indicator, which may help comprehend significant nodes in the trend in this field. Nonetheless, this study is bound to have some limitations. To begin with, we counted only English articles and reviews from SCI-Expanded-indexed journals. Second, some details may be omitted due to VOSviewer’s inability to analyze the full text of publications. Finally, some newly published excellent papers with low NC may be excluded due to lag. We hope that future studies will look at more databases and get a complete picture of how macrophage-related RA is studied around the world.

## Conclusions

This study used bibliometric analysis to summarized the articles on macrophages-related RA. It shed light on evolution of publications and their citations of macrophages-related RA over the last 22 years. The number of articles on macrophages-related RA were increasing. Clinical studies or clinical guidelines published in high-impact journals received a higher rate of citations in RA. The discovery of new targeted therapy drugs, exploration of therapeutic mechanism and drug delivery will remain the focus of future research. We hope this bibliometric analysis provides a beneficial reference to researchers to better comprehend the current state of macrophage-related RA research from a macro viewpoint.

## Data availability statement

The original contributions presented in the study are included in the article/supplementary material. Further inquiries can be directed to the corresponding author.

## Author contributions

YLX and ZXC conceived and designed the experiments. YLX and JLH performed the experiments. YLX and ZMZ wrote the paper. YLX and ZMZ contributed equally to this work. All authors contributed to the article and approved the submitted version.

## Funding

This work was supported in part by the Natural Science Foundation of Zhejiang Province (Grants LQ22H280020)

## Conflict of interest

The authors declare that the research was conducted in the absence of any commercial or financial relationships that could be construed as a potential conflict of interest.

## Publisher’s note

All claims expressed in this article are solely those of the authors and do not necessarily represent those of their affiliated organizations, or those of the publisher, the editors and the reviewers. Any product that may be evaluated in this article, or claim that may be made by its manufacturer, is not guaranteed or endorsed by the publisher.

## References

[1] Boutet MA Courties G Nerviani A Le Goff B Apparailly F Pitzalis C . Novel insights into macrophage diversity in rheumatoid arthritis synovium. Front Immunol (2020) 11:1060. doi: 10.3389/fimmu.2020.01060 33476818

[2] Scherer HU Häupl T Burmester GR . The etiology of rheumatoid arthritis. J Autoimmune (2020) 10:102400. doi: 10.1016/j.jaut.2019.102400 31980337

[3] Chaichian Y Genovese MC Weisman MH . The road to rheumatoid arthritis prevention: Challenges and opportunities. Clin Rheumatol (2020) 39:1379–81. doi: 10.1007/s10067-020-05016-4 32170486

[4] Drosos AA Pelechas E Kaltsonoudis E Voulgari PV . Therapeutic options and cost-effectiveness for rheumatoid arthritis treatment. Curr Rheumatol Rep (2020) 22:44. doi: 10.1007/s11926-020-00921-8 32591916

[5] Zhang A Lee YC . Mechanisms for joint pain in rheumatoid arthritis (RA): From cytokines to central sensitization. Curr Osteoporos Rep (2018) 16(5):603–10. doi: 10.1007/s11914-018-0473-5 PMC6157001 30128836

[6] Greenblatt MB Tsai JN Wein MN . Bone turnover markers in the diagnosis and monitoring of metabolic bone disease. Clin Chem (2017) 63:464–74. doi: 10.1373/clinchem.2016.259085 PMC5549920 27940448

[7] Murray PJ . Macrophage polarization. Annu Rev Physiol (2017) 79:541–66. doi: 10.1146/annurev-physiol-022516-034339 27813830

[8] Orsi M Palmai-Pallag M Yakoub Y Ibouraadaten S De Beukelaer M Bouzin C . Monocytic ontogeny of regenerated macrophages characterizes the mesothe liomagenic responses to carbon nanotubes. Front Immunol (2021) 12:666107. doi: 10.3389/fimmu.2021.666107 34194430 PMC8236701

[9] Ma Q . Polarization of immune cells in the pathologic response to inhaled particulates. Front Immunol (2020) 11:1060. doi: 10.3389/fimmu.2020.01060 32625201 PMC7311785

[10] Udalova IA Mantovani A Feldmann M . Macrophage heterogeneity in the context of rheumatoid arthritis. Nat Rev Rheumatol (2016) 12:472–85. doi: 10.1038/nrrheum 27383913

[11] Li R Sun J Hu H Zhang Q Sun R Zhou S . Research trends of acupuncture therapy on knee osteoarthritis from 2010 to 2019: A bibliometric analysis. J Pain Res (2020) 13:1901–13. doi: 10.2147/JPR.S258739 PMC7394582 32801848

[12] Luo H Cai Z Huang Y Song J Ma Q Yang X . Study on pain catastrophizing from 2010 to 2020: A bibliometric analysis *via* CiteSpace. Front Psychol (2021) 12:759347. doi: 10.3389/fpsyg.2021.759347 34975649 PMC8718514

[13] Landis JR Koch GG . The measurement of observer agreement for categorical data. Biometrics (1977) 33:159–74. doi: 10.2307/2529310 843571

[14] Yu Y Li Y Zhang Z . A bibliometric analysis using VOSviewer of publications on COVID-19. Ann Trans Med (2020) 8(13):816. doi: 10.21037/atm-20-4235 PMC7396244 32793661

[15] Chen C . Searching for intellectual turning points: progressive knowledge domain visualization. Proc Natl Acad Sci USA (2004) 101(Suppl 1):5303–10. doi: 10.1073/pnas.0307513100 PMC387312 14724295

[16] González-Alcaide G Calafat A Becoña E Thijs B Glänzel W . Co-Citation analysis of articles published in substance abuse journals: Intellectual structure and research fields (2001-2012). J Stud Alcohol Drugs (2016) 77:710–22. doi: 10.15288/jsad.2016.77.710 27588529

[17] Keane J Gershon S Wise RP Mirabile-Levens E Kasznica J Schwieterman WD . Tuberculosis associated with infliximab, a tumor necrosis factor alpha-neutralizing agent. N Engl J Med (2001) 345:1098–104. doi: 10.1056/NEJMoa011110 11596589

[18] Deshmane SL Kremlev S Amini S Sawaya BE . Monocyte chemoattractant protein-1 (MCP-1): An overview. J Interferon Cytokine Res (2009) 29:313–26. doi: 10.1089/jir.2008.0027 PMC2755091 19441883

[19] Lawrence T . The nuclear factor NF-kappa b pathway in inflammation. Cold Spring Harb Perspect Biol (2009) 1:a001651. doi: 10.1101/cshperspect.a001651 20457564 PMC2882124

[20] Kleinberg J . Bursty and hierarchical structure in streams. Data Min Knowl Disc. (2003) 7:373–97. doi: 10.1023/A:1024940629314

[21] McInnes IB Schett G . Cytokines in the pathogenesis of rheumatoid arthritis. Nat Rev Immunol (2007) 7(6):429–42. doi: 10.1038/nri2094 17525752

[22] McInnes IB Buckley CD Isaacs JD . Cytokines in rheumatoid arthritis - shaping the immunological landscape. Nat Rev Rheumatol (2016) 12(1):63–8. doi: 10.1038/nrrheum.2015.171 26656659

[23] McInnes IB Schett G . The pathogenesis of rheumatoid arthritis. N Engl J Med (2011) 365(23):2205–19. doi: 10.1056/NEJMra1004965 22150039

[24] Liu Y Chen H Chen Z Qiu J Pang H Zhou Z . Novel roles of the Tim family in immune regulation and autoimmune diseases. Front Immunol (2021) 12:748787. doi: 10.3389/fimmu.2021.748787 34603337 PMC8484753

[25] Angelotti F Parma A Cafaro G Capecchi R Alunno A Puxeddu I . One year in review 2017: Pathogenesis of rheumatoid arthritis. Clin Exp Rheumatol (2017) 35(3):368–78 Available at: https://www.clinexprheumatol.org/abstract.asp?a=14254. 28631608

[26] Huang Q Pope RM . Toll-like receptor signaling: A potential link among rheumatoid arthritis, systemic lupus, and atherosclerosis. J Leukoc Biol (2010) 88:253–62. doi: 10.1189/jlb.0310126 PMC2908942 20484668

[27] Cuda CM Pope RM Perlman H . The inflammatory role of phagocyte apoptotic pathways in rheumatic diseases. Nat Rev Rheumatol (2016) 12:543–58. doi: 10.1038/nrrheum.2016.132 PMC5297631 27549026

[28] Mills CD . Anatomy of a discovery: m1 and m2 macrophages. Front Immunol (2015) 6:212. doi: 10.3389/fimmu.2015.00212 25999950 PMC4419847

[29] Arango Duque G Descoteaux A . Macrophage cytokines: Involvement in immunity and infectious diseases. Front Immunol (2014) 5:491. doi: 10.3389/fimmu.2014.00491 25339958 PMC4188125

[30] Mills CD . M1 and M2 macrophages: Oracles of health and disease. Crit Rev Immunol (2012) 32:463–88. doi: 10.1615/critrevimmunol.v32.i6.10 23428224

[31] Cutolo M Campitiello R Gotelli E Soldano S . The role of M1/M2 macrophage polarization in rheumatoid arthritis synovitis. Front Immunol (2022) 13:867260. doi: 10.3389/fimmu.2022.867260 35663975 PMC9161083

[32] Akram M Daniyal M Sultana S Owais A Akhtar N Zahid R . Traditional and modern management strategies for rheumatoid arthritis. Clin Chim Acta (2021) 512:142–55. doi: 10.1016/j.cca.2020.11.003 33186593

[33] Parameswaran N Patial S . Tumor necrosis factor-α signaling in macrophages. Crit Rev Eukaryot Gene Expr (2010) 20(2):87–103. doi: 10.1615/critreveukargeneexpr.v20.i2.10 21133840 PMC3066460

[34] Nam J Emery P . Aspects of TNF inhibitor therapy in rheumatoid arthritis. Mod Rheumatol (2010) 20(4):325–30. doi: 10.1007/s10165-010-0277-7 20195684

[35] Li SJ Perez-Chada LM Merola JF . TNF inhibitor-induced psoriasis: Proposed algorithm for treatment and management. J Psoriasis Psoriatic Arthritis (2019) 4:70–80. doi: 10.1177/2475530318810851 31093599 PMC6513344

[36] Yamanaka H . TNF as a target of inflammation in rheumatoid arthritis. Endocr Metab Immune Disord Drug Targets (2015) 15:129–34. doi: 10.2174/1871530315666150316121808 25772178

[37] Srivastava S Rasool M . Underpinning IL-6 biology and emphasizing selective JAK blockade as the potential alternate therapeutic intervention for rheumatoid arthritis. Life Sci (2022) 298:120516. doi: 10.1016/j.lfs.2022.120516 35367240

[38] Fang H Sha Y Yang L Jiang J Yin L Li J . Macrophage-targeted hydroxychloroquine nanotherapeutics for rheumatoid arthritis therapy. ACS Appl Mater Interfaces (2022) 14(7):8824–37. doi: 10.1021/acsami.1c23429 35156814

[39] George MD Baker JF Winthrop K Hsu JY Wu Q Chen L . Risk for serious infection with low-dose glucocorticoids in patients with rheumatoid arthritis. A cohort study. Ann Intern Med (2020) 173:870–8. doi: 10.7326/M20-1594 PMC8073808 32956604

[40] Wang S Lv J Meng S Tang J Nie L . Recent advances in nanotheranostics for treat-to-Target of rheumatoid arthritis. Adv Healthc Mater (2020) 9(6):e1901541. doi: 10.1002/adhm.201901541 32031759

[41] Yang Y Guo L Wang Z Liu P Liu X Ding J . Targeted silver nanoparticles for rheumatoid arthritis therapy *via* macrophage apoptosis and polarization. Biomaterials (2021) 264:120390. doi: 10.1016/j.biomaterials.2020.120390 32980634

[42] Li R He Y Zhu Y Jiang L Zhang S Qin J . Route to rheumatoid arthritis by macrophage-derived microvesicle-coated nanoparticles. Nano Lett (2019) 19(1):124–34. doi: 10.1021/acs.nanolett.8b03439 30521345

[43] Anita C Munira M Mural Q Shaily L . Topical nanocarriers for management of rheumatoid arthritis: A review. BioMed Pharmacother (2021) 141:111880. doi: 10.1016/j.biopha.2021.111880 34328101

[44] Jeong M Park JH . Nanomedicine for the treatment of rheumatoid arthritis. Mol Pharm (2021) 18:539–49. doi: 10.1021/acs.molpharmaceut.0c00295 32502346

