# Supplementary material for: Immune Effects of Macrophages in Rheumatoid Arthritis: A Bibliometric Analysis From 2000 to 2021
Source: Front Immunol. 2022 Sep 12;13:903771. doi: 10.3389/fimmu.2022.903771 (PMC9510364; doi:10.3389/fimmu.2022.903771)
Supplement: Supplementary file 1 [file DataSheet_1.docx]

**Supplementary Material**

**Immune Effects of Macrophages in Rheumatoid Arthritis: A Bibliometric Analysis from 2000 to 2021**

Yunling Xu ^1†^, Zhongmin Zhang ^2†^, Jiaolong He ^3^, Zhenxing Chen ^2*^

*^1^ Department of Basic Medical, Zhejiang Academy of Traditional Chinese Medicine, Hangzhou 310007, China*

*^2^ College of Pharmacy, Guangxi University of Chinese Medicine, Nanning 530200, China*

*^3^ Department of Intensive Care, First Affiliated Hospital of Jishou University, Jishou, 416000, China.*

† Yunling Xu and Zhongmin Zhang contributed equally to this work and should be considered co-first authors.

* Correspondence: 416158716@qq.com (Zhenxing Chen); Tel.: +86-771-4953513


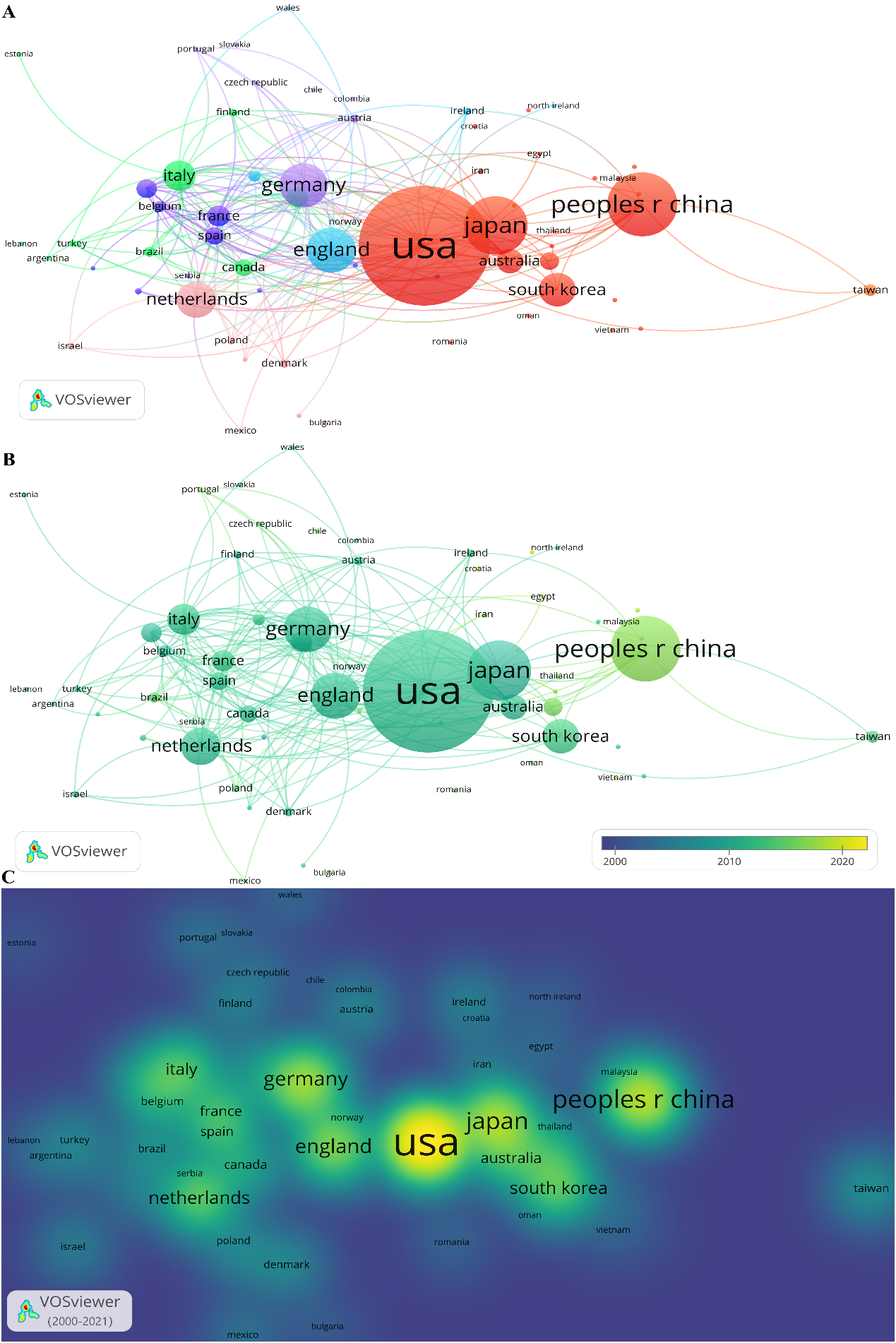


**Supplementary Figure 1**| Countries/regions co-authorship analysis from 2000-2021. (**A**) Cluster analysis of publications from different countries/regions. (**B**) Evolution of publications from different countries/regions. (**C**) Evolution of publications from different countries/regions frequency.
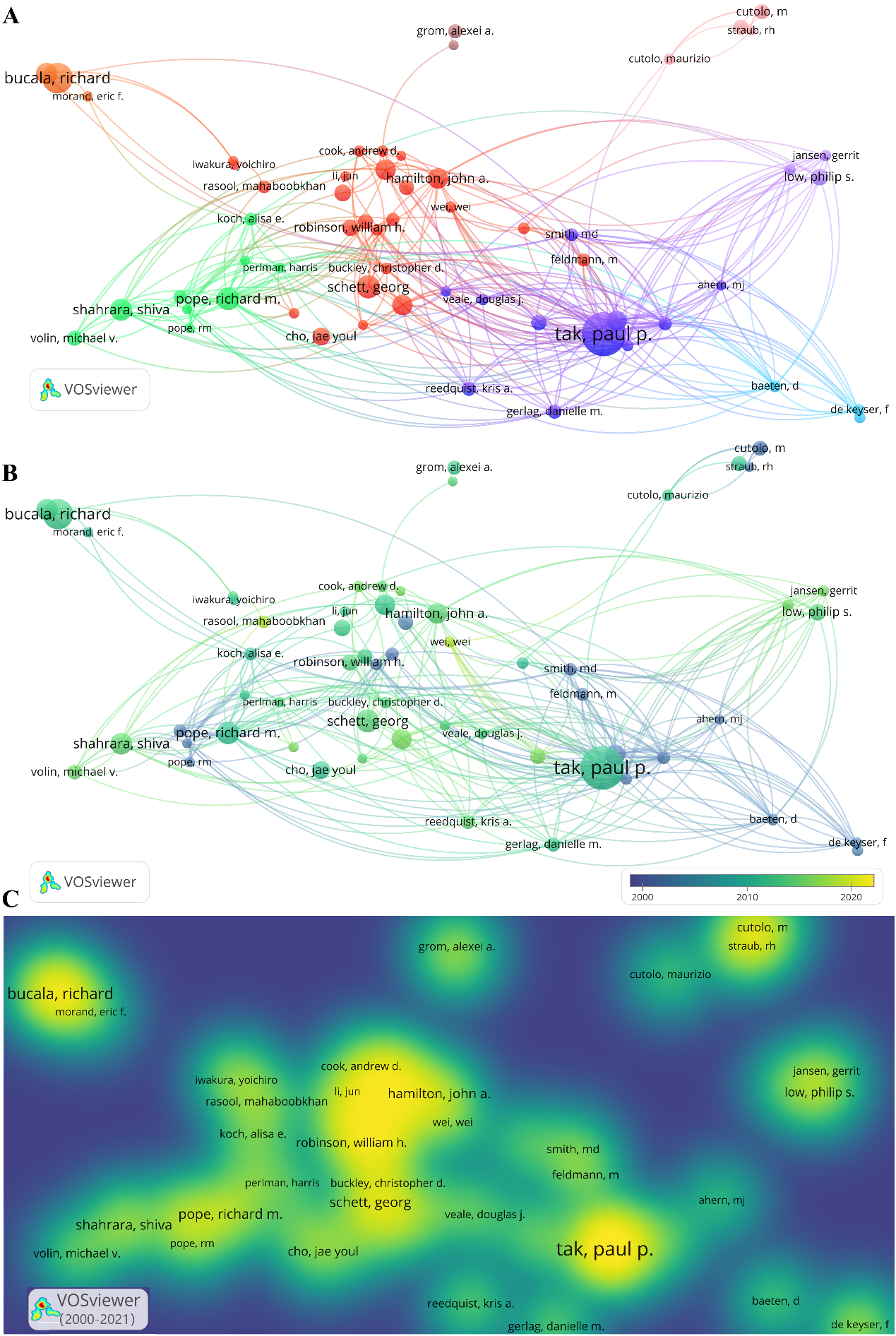
 **Supplementary Figure** **2**| Author co-authorship analysis from 2000-2021.
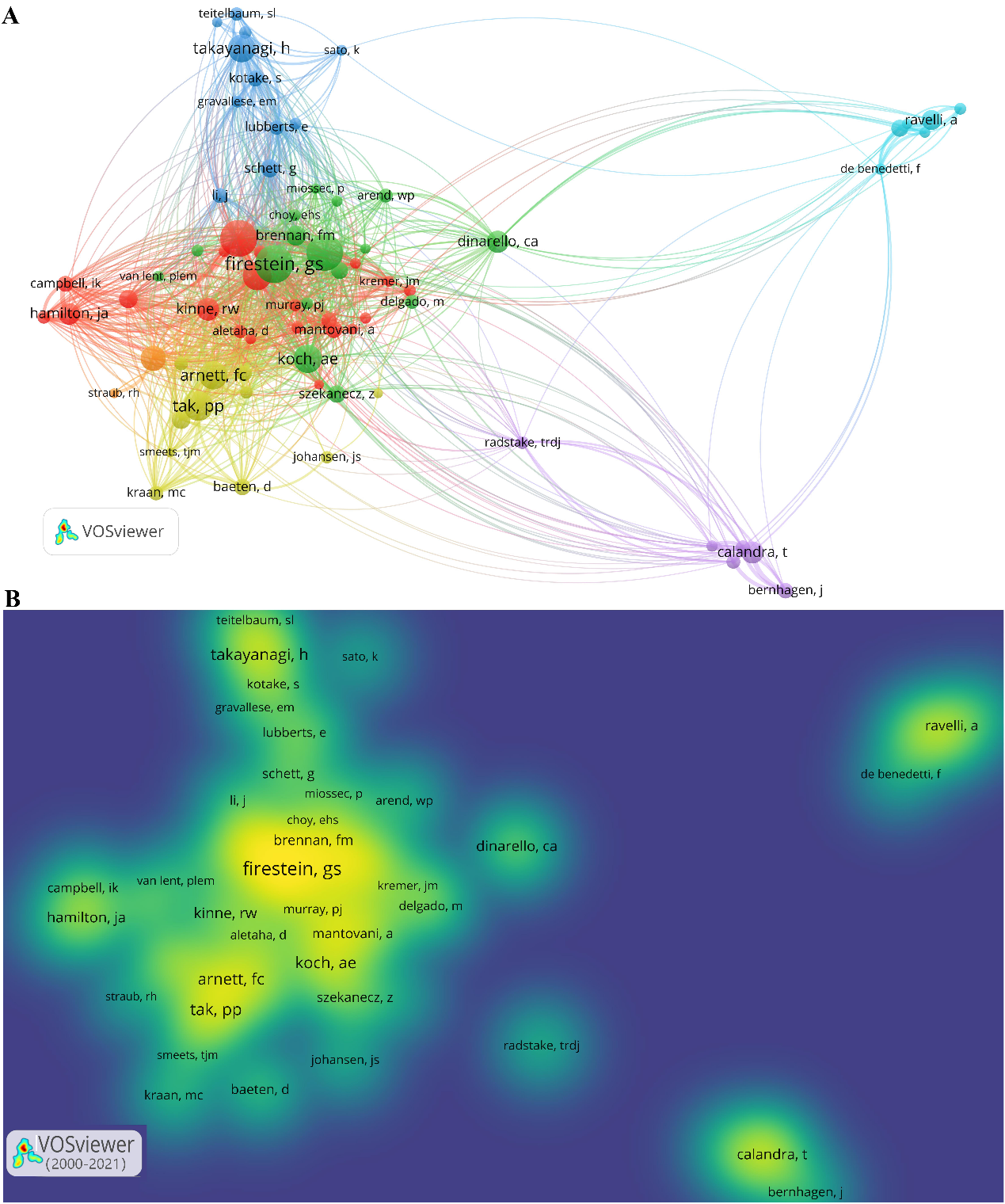
 **Supplementary Figure 3** **|** Author co-citation network visualization map from 2000-2021. (**A**) Cluster analysis of co-citation author. (**B**) Evolution of co-citation author frequency.


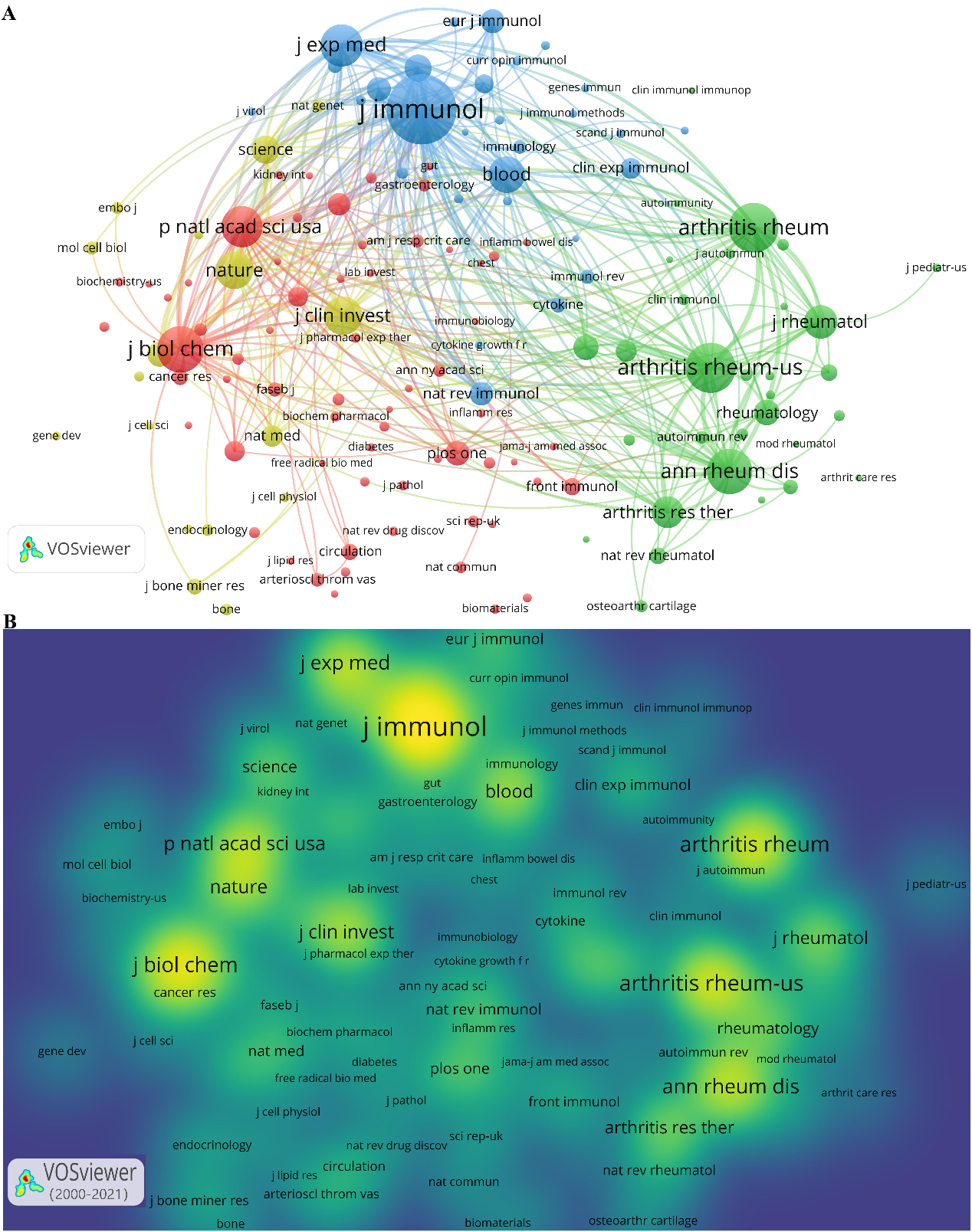
 **Supplementary Figure 4 |** Journals co-citation network visualization map from 2000-2021. (**A**) Cluster analysis of co-citation journals. (**B**) Evolution of co-citation journals frequency.


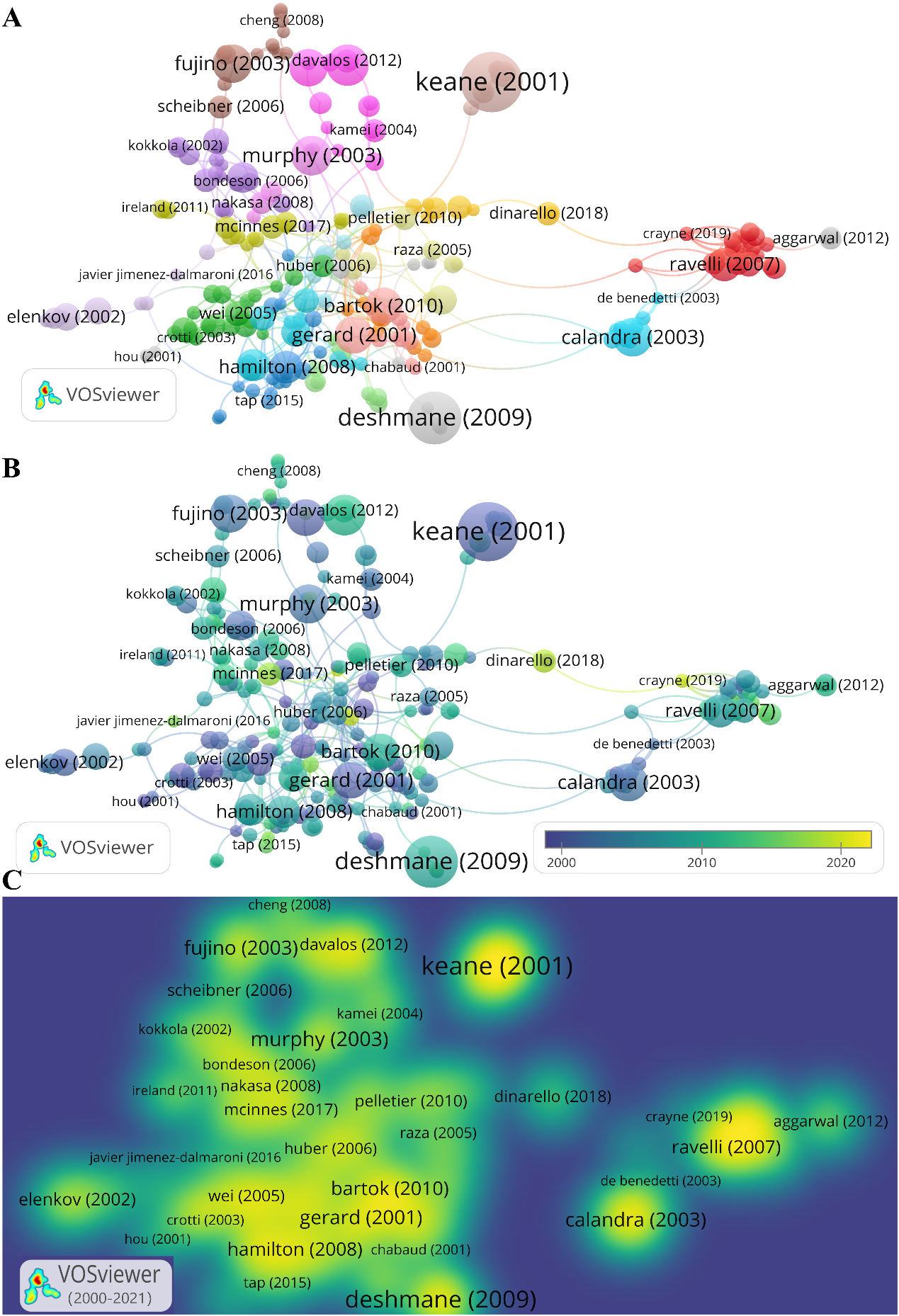
 **Supplementary Figure 5 |** Citations document network visualization map from 2000-2021. (A) Cluster analysis of citations document. (**B**) Evolution of citations document. (**C**) Evolution of citations document frequency.


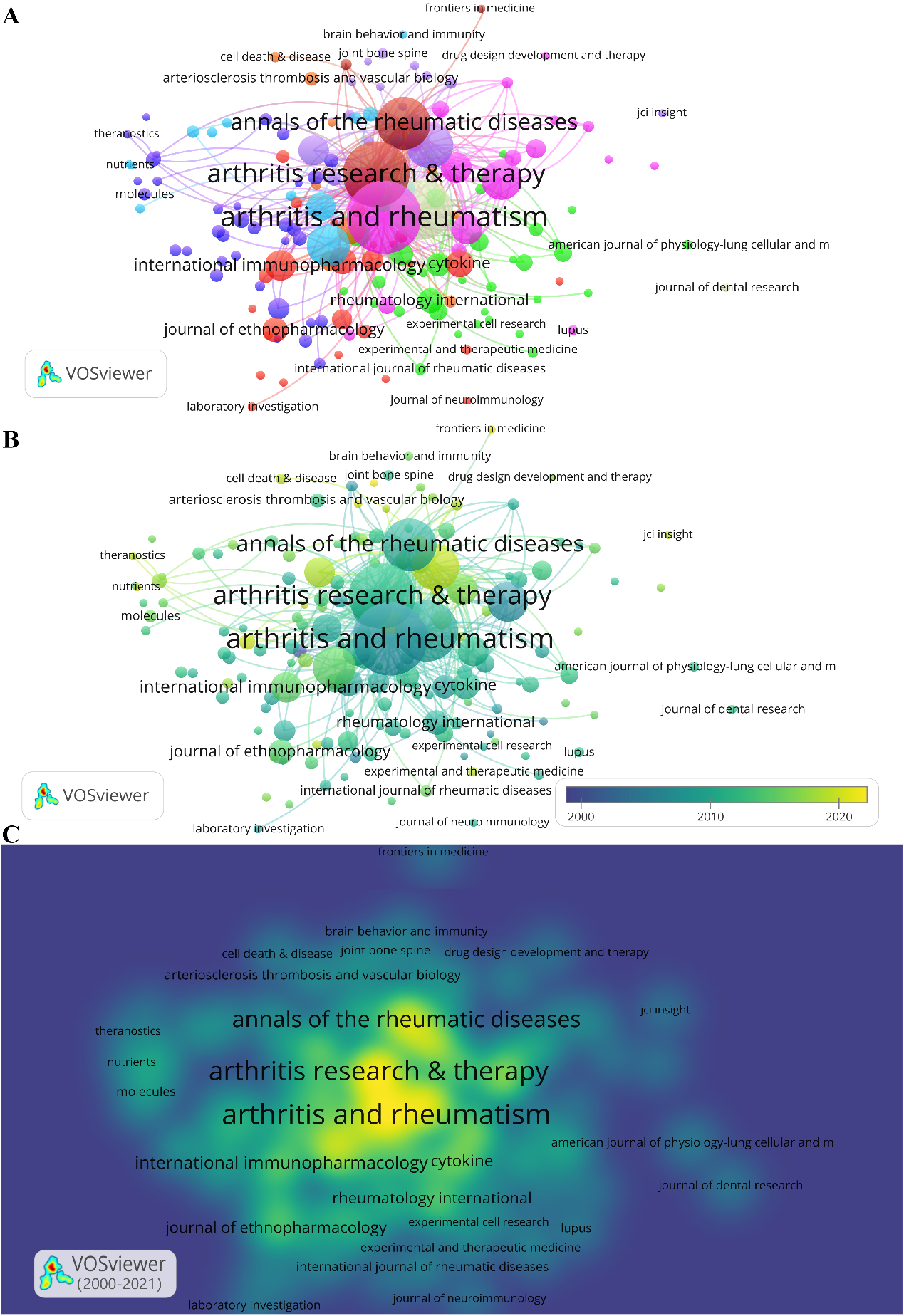
 **Supplementary Figure 6 |** Citation journals analysis from 2000-2021. (A) Cluster analysis of citation journals. (**B**)Evolution of citation journals. (**C**) Evolution of citation journals frequency.

**Supplementary Table 1 | The publications on macrophage-related RA and other immune cells.**

| **Rank** | **RA-related topic** | **NP** | **NC** | **AC** | **H-index** |
| --- | --- | --- | --- | --- | --- |
| 1 | Macrophage OR Macrophages | 7253 | 311601 | 46.53 | 208 |
| 2 | (Macrophage OR macrophages) **AND** (B-lymphocyte OR B cell OR B-cell) | 3248 | 170497 | 54.3 | 173 |
| 3 | (Macrophage OR macrophages) **AND** (T-lymphocyte OR T cell OR T-cell) | 3018 | 177478 | 60.91 | 183 |
| 4 | (Macrophage OR macrophages) **AND** (dendritic cell OR Dendritic cells OR DC) | 1197 | 68654 | 58.43 | 127 |
| 5 | (Macrophage OR macrophages) **AND** (natural-killer-cell OR natural killer cell OR NK) | 360 | 22300 | 63.14 | 80 |
| 6 | (Macrophage OR macrophages) **AND** (Mast Cell OR Mast cells OR MCs) | 223 | 15093 | 68.09 | 69 |

**Supplementary Table 2 |** The publications on macrophage-related RA treatment target.

| **Rank** | **Retrieve Keywords** | **NP** | **NC** | **AC** | **H-index** |
| --- | --- | --- | --- | --- | --- |
| 1 | (Rheumatoid arthritis treatment OR RA-treatment OR rheumatoid arthritis therapeutic target) **AND** (Macrophage OR Macrophages) **AND** (tumor-necrosis-factor OR Tumor necrosis factor OR TNF) | 1275 | 64055 | 51.35 | 116 |
| 2 | (Rheumatoid arthritis treatment OR RA-treatment OR rheumatoid arthritis therapeutic target) **AND** (Macrophage OR Macrophages) **AND** (interleukin 1 OR IL-1) | 786 | 35622 | 46.16 | 96 |
| 3 | (Rheumatoid arthritis treatment OR RA-treatment or rheumatoid arthritis therapeutic target) **AND** (Macrophage OR Macrophages) **AND** (interleukin 6 OR IL-6) | 654 | 29665 | 45.97 | 85 |
| 4 | (Rheumatoid arthritis treatment OR RA-treatment or rheumatoid arthritis therapeutic target) **AND** (Macrophage OR Macrophages) **AND** (interleukin 10 OR IL-10) | 279 | 14278 | 51.46 | 61 |
| 5 | (Rheumatoid arthritis treatment OR RA-treatment or rheumatoid arthritis therapeutic target) **AND** (Macrophage OR Macrophages) **AND** (interleukin 17 OR IL-17) | 185 | 11474 | 62.56 | 58 |
| 6 | (Rheumatoid arthritis treatment OR RA-treatment or rheumatoid arthritis therapeutic target) **AND** (Macrophage OR Macrophages) **AND** (Granulocyte Macrophage Colony Stimulating Factor OR GM-CSF) | 179 | 9499 | 55.76 | 55 |
| 7 | (Rheumatoid arthritis treatment OR RA-treatment or rheumatoid arthritis therapeutic target) **AND** (Macrophage OR Macrophages) **AND** (Macrophage OR Macrophages) **AND** (interferon-gamma OR IFN-γ) | 147 | 7438 | 50.95 | 53 |
| 8 | (Rheumatoid arthritis treatment OR RA-treatment or rheumatoid arthritis therapeutic target) **AND** (Macrophage OR Macrophages) **AND** (interleukin 12 OR IL-12) | 122 | 6383 | 52.48 | 44 |
| 9 | (Rheumatoid arthritis treatment OR RA-treatment or rheumatoid arthritis therapeutic target) **AND** (Macrophage OR Macrophages) **AND** (cyclooxygenase-2 OR COX2) | 94 | 3496 | 37.33 | 32 |
| 10 | (Rheumatoid arthritis treatment OR RA-treatment or rheumatoid arthritis therapeutic target) **AND** (Macrophage OR Macrophages) **AND** (interleukin 18 OR IL-18) | 85 | 4442 | 52.92 | 40 |
| 11 | (Rheumatoid arthritis treatment OR RA-treatment or rheumatoid arthritis therapeutic target) **AND** (Macrophage OR Macrophages) **AND** (interleukin 23 OR IL-23) | 60 | 3278 | 54.83 | 32 |
| 12 | (Rheumatoid arthritis treatment OR RA-treatment or rheumatoid arthritis therapeutic target) **AND** (Macrophage OR Macrophages) **AND** (Janus Kinase 1 OR JAK 1) | 31 | 1170 | 37.9 | 19 |
| 13 | (Rheumatoid arthritis treatment OR RA-treatment or rheumatoid arthritis therapeutic target) **AND** (Macrophage OR Macrophages) **AND** (Janus Kinase 2 OR JAK 2) | 20 | 704 | 35.3 | 14 |
| 14 | (Rheumatoid arthritis treatment OR RA-treatment or rheumatoid arthritis therapeutic target) **AND** (Macrophage OR Macrophages) **AND** (Janus Kinase 3 OR JAK 3) | 18 | 688 | 38.33 | 12 |
| 15 | (Rheumatoid arthritis treatment OR RA-treatment or rheumatoid arthritis therapeutic target) **AND** (Macrophage OR Macrophages) **AND** (p38MAPK) | 10 | 418 | 41.9 | 7 |
